# Two cases of symptomatic adventitial cystic disease involving the popliteal artery and femoral vein

**DOI:** 10.1016/j.jvscit.2026.102216

**Published:** 2026-03-02

**Authors:** Shota Inoue, Atsuhiko Sato, Mitsuru Yokoyama, Kei Kono, Yutaka Takazawa, Shigefumi Matsuyama

**Affiliations:** a Department of Cardiovascular Surgery, Toranomon Hospital, Tokyo, Japan; b Department of Pathology, Toranomon Hospital, Tokyo, Japan

**Keywords:** Adventitial cystic disease, Popliteal artery, Femoral vein, Vascular surgery

## Abstract

Adventitial cystic disease is a rare condition involving mucinous cysts within the adventitial layer of arteries or veins. We report two distinct cases: one in the popliteal artery presenting with intermittent claudication and another in the femoral vein presenting with limb swelling and pain. The diagnosis was established based on characteristic imaging findings, and both lesions were completely excised without recurrence. The arterial cyst contained clear gelatinous fluid, and the venous cyst contained hemorrhagic material, possibly related to anticoagulant use. Adventitial cystic disease should be considered in the differential diagnosis of peripheral vascular conditions owing to its variable presentation.

Adventitial cystic disease (ACD) is an uncommon vascular disorder characterized by cyst formation within the adventitial layer of the vessel wall, accounting for approximately 0.1% of all vascular pathologies.^1^ The condition predominantly affects arteries; venous involvement is uncommon. In this report, we describe two cases of ACD that developed at distinct vascular sites, the popliteal artery and the femoral vein, each demonstrating unique clinical features and cystic characteristics. This comparative report aimed to highlight the diverse clinical presentations, diagnostic findings, and surgical management of ACD. Written informed consent was obtained from the patients for publication.

## Case 1

**Fig 1:**
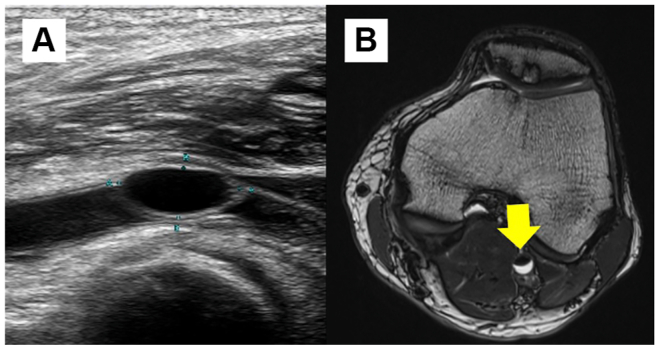
Preoperative imaging of the popliteal artery adventitial cystic disease (ACD). **(A)** Duplex ultrasound image showing a cystic lesion compressing the arterial lumen. **(B)** Magnetic resonance imaging demonstrating a high T2-weighted and low T1-weighted signal cyst surrounding the popliteal artery (*arrow*).

**Fig 2:**
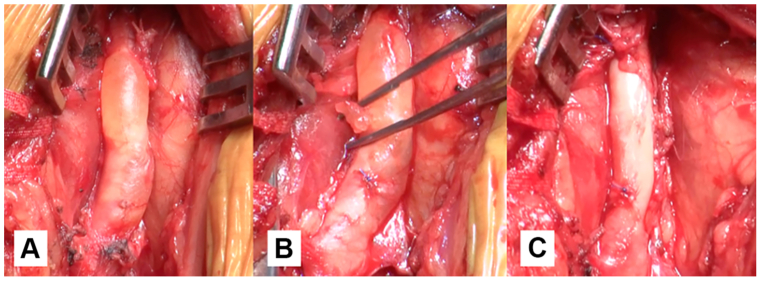
Intraoperative findings of the popliteal artery adventitial cystic disease (ACD). **(A)** A cystic lesion 14 mm in diameter is identified on the adventitia of the popliteal artery. **(B)** The cyst contains clear gelatinous material. **(C)** Complete excision performed without leaving the cyst wall.

A 65-year-old man presented with intermittent claudication in his left leg. His medical history included hypertension, dyslipidemia, exertional angina, nonfunctioning pituitary adenoma, and autosomal-dominant polycystic kidney disease. On the left side, the ankle-brachial index was 0.56. Contrast-enhanced computed tomography (CT) scan revealed significant stenosis of the popliteal artery. However, subsequent angiography revealed no definite luminal narrowing. Duplex ultrasound examination identified a cystic lesion compressing the lumen of the left popliteal artery (Fig 1, *A*). Magnetic resonance imaging revealed a cyst at the same location, exhibiting high T2-weighted and low T1-weighted signal intensities (Fig 1, *B*). Based on these characteristic imaging findings, ACD of the popliteal artery was suspected, and surgical excision was performed via a posterior approach with the patient in the prone position (Fig 2). The excised cyst contained clear gelatinous material, and histopathological examination revealed a multilocular structure with Alcian blue-positive mucin. Postoperatively, the patient's symptoms resolved completely, and the ankle-brachial index returned to normal. The patient has remained free of recurrence for 7 months.

## Case 2

**Fig 3:**
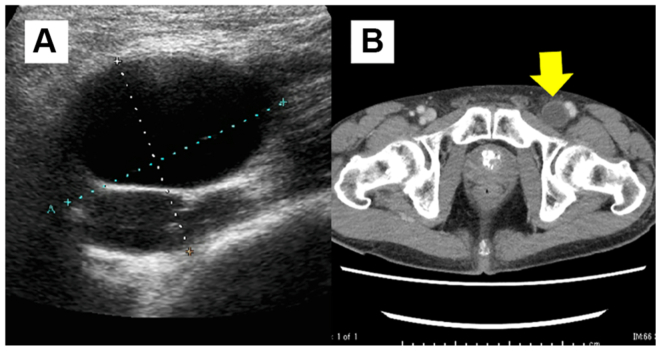
Preoperative imaging of the femoral vein adventitial cystic disease (ACD). **(A)** Duplex ultrasound image showing a multilocular cystic lesion adjacent to the femoral vein. **(B)** Contrast-enhanced computed tomography (CT) scan confirming a cystic mass compressing the vein (*arrow*).

**Fig 4:**
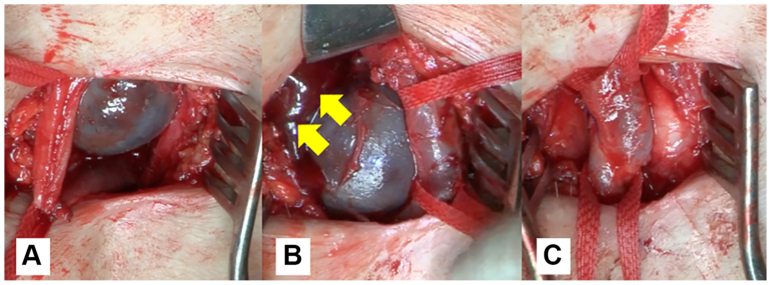
Intraoperative findings of the femoral vein adventitial cystic disease (ACD). **(A)** A cystic lesion 30 mm in diameter compressing the femoral vein with distal venous dilatation. **(B)** Cyst contains hemorrhagic gelatinous material (*arrow*). **(C)** Cyst completely excised without leaving the wall.

**Fig 5:**
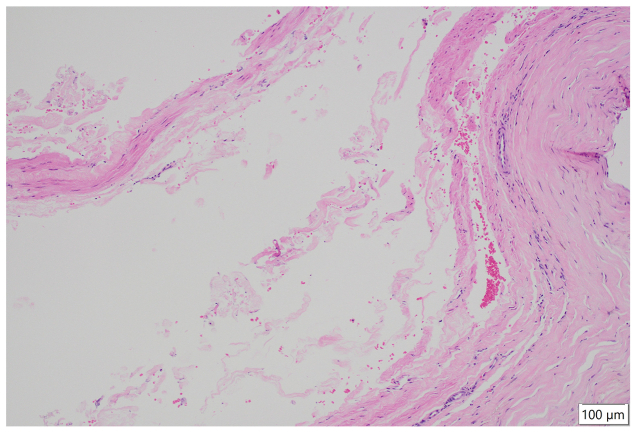
Histopathological findings of femoral vein adventitial cystic disease (ACD) (case 2). Hematoxylin and eosin stain (original magnification ×100) demonstrating a cystic structure composed of collagenous fibrous tissue containing erythrocytes.

An 80-year-old man presented with swelling and pain in his left lower limb. His medical history included atrial fibrillation, for which he was receiving a direct oral anticoagulant (DOAC). Duplex ultrasound examination revealed a cystic lesion encasing the left femoral vein (Fig 3, *A*), and contrast-enhanced CT scan confirmed a multilocular cyst at the same site (Fig 3, *B*). Based on these imaging findings, femoral vein ACD was suspected, and surgical excision was performed (Fig 4). The cyst contained a hemorrhagic gelatinous material, and histopathological examination revealed a cystic structure composed of collagenous fibrous tissue containing erythrocytes (Fig 5). Postoperatively, the limb edema resolved promptly and no recurrence was observed over 21 months of follow-up.

## Discussion

ACD is a rare vascular disorder characterized by cyst formation within the adventitial layer of the vessel wall,^2^ although its underlying pathogenesis remains unclear. Because clinical presentation varies according to the site of involvement, understanding its diverse manifestations is essential, and ACD should be considered in the differential diagnosis of peripheral vascular diseases.

The pathogenesis of ACD remains unclear, with several hypotheses proposed to explain its origin. These include the trauma theory, which attributes the condition to repetitive mechanical stress such as trauma leading to cystic degeneration of the vascular adventitia; the synovial theory, which suggests that synovial cysts from adjacent joint capsules extend into the vessel wall; the developmental theory, which posits that mucin-secreting synovial cells become aberrantly implanted within the adventitia during embryogenesis; and the systemic disorder theory, which proposes a possible association with connective tissue diseases.^3^ Currently, none of these theories alone adequately explains all reported cases, and multiple mechanisms have been suggested to act in combination to cause the disease.^4^

A distinctive feature of the second case was the presence of a hemorrhagic cyst, rather than the clear, gelatinous fluid typically seen in ACD.^5^ The patient was receiving DOAC for atrial fibrillation. According to the trauma theory, repeated shear stress can lead to microscopic dissections and intramural hemorrhage between the media and adventitia, which may subsequently evolve into cystic cavities.^6^ The anticoagulant effect of DOAC therapy may have exacerbated these microhemorrhages, producing the macroscopic appearance of a hemorrhagic cyst. This finding supports the trauma theory as a possible mechanism. Nevertheless, secondary bleeding from neovascularization within a preexisting cyst wall, influenced by anticoagulant therapy, cannot be excluded. Clinically, when a cystic lesion surrounding a vessel is identified in patients receiving anticoagulant therapy, ACD should be included in the differential diagnosis.

Arterial ACD constitutes approximately 95% of all ACD cases, with the popliteal artery being the most commonly affected vessel, representing nearly 87% of all arterial cases.^7^ The typical clinical manifestation is intermittent claudication, as observed in patient 1, which underscores the importance of considering ACD in the differential diagnosis of lower limb ischemia. On imaging, contrast-enhanced CT scan or angiography typically demonstrate the characteristic hourglass-shaped luminal narrowing caused by concentric external compression; however, in some cases, no apparent stenosis is observed.^4^ In the first case, diagnosis proved challenging. Owing to the patient's advanced age, presentation with intermittent claudication, and decreased ankle-brachial index, arteriosclerosis obliterans (ASO) was initially suspected. Although contrast-enhanced CT scan revealed focal narrowing of the popliteal artery, angiography revealed no definite stenosis. Furthermore, subsequent duplex ultrasound examination and magnetic resonance imaging identified a cystic lesion compressing the artery, raising the suspicion of ACD. These findings indicate that the degree of arterial compression in ACD can vary depending on the body position or limb movement, potentially resulting in functional ischemia during exercise.^8^

This diagnostic course also highlights important considerations. Because ASO was initially suspected owing to its much higher prevalence, ACD was not considered at the time of angiography, and consequently, intravascular ultrasound was not performed. However, intravascular ultrasound is well-suited for delineating cystic lesions and may aid in diagnosing ACD in selected cases.^9^ Moreover, dynamic maneuvers during angiography may detect functional arterial compression not apparent on static imaging. Furthermore, ultrasound examination, a minimally invasive modality, is useful for diagnosing both ASO and ACD and should be considered early in the evaluation of peripheral vascular disease.

Venous ACD is rare and accounts for approximately 5% of all ACD cases.^7^ A 2021 literature review identified only 72 reported cases worldwide, highlighting its rarity.^10^ The most commonly affected vessel is the common femoral vein, representing approximately 65% of venous cases. The typical clinical manifestation is lower limb edema, as observed in patient 2, necessitating differentiation from deep vein thrombosis or neoplastic lesions. In this case, the patient was receiving anticoagulant therapy, and the cyst appeared hemorrhagic and gelatinous. Although ACD typically contain clear,^5^ mucinous material, this atypical presentation may suggest a possible association with anticoagulant use.

The standard treatment for ACD is complete cyst excision, with or without vascular reconstruction. Because residual cyst wall tissue may cause recurrence, aspiration or drainage alone is associated with a high likelihood of recurrence. A literature review of venous ACD reported recurrence in all 10 patients treated with aspiration and both patients treated with drainage alone.^10^ In both cases, surgical excision led to significant symptom improvement and no recurrence, leading to improved activities of daily living.

## Conclusions

ACD is an extremely rare condition that can affect both arteries and veins. Because its symptoms vary depending on the site of involvement, ACD should always be considered in the differential diagnosis of peripheral vascular diseases. Early diagnosis and complete surgical excision of the cyst wall are essential to achieve favorable outcomes.

## Funding

None.

## Disclosures

None.
